# PReS13-SPK-1277: Role of ß2GPI in APS

**DOI:** 10.1186/1546-0096-11-S2-I12

**Published:** 2013-12-05

**Authors:** PP De Groot

**Affiliations:** 1 University Medical Center Utrecht, Utrecht, The Netherlands

## 

b_2_-Glycoprotein I (b_2_-GPI), also known as apolipoprotein H, is a 50 kDa protein that was described for the first time in 1961, and in 1968 the first apparently healthy person was identified deficient in this protein. As no function could be attributed to b_2_-GPI, the protein did not receive much attention and on average a single publication per year appeared in the literature. From 1990 onwards, the interest in this apparently obsolete protein has increased significantly when b_2_-GPI was identified as the most important antigen in an autoimmune disease called the antiphospholipid syndrome (APS). APS is an auto-immune disease characterized by thrombotic complications in both arteries and veins as well as pregnancy-related complications in combination with the presence of so-called antiphospholipid antibodies in the plasma of these patients. It is now generally accepted that these auto-antibodies are not directed against negatively charged phospholipids but towards proteins bound to these phospholipids. Animal studies have shown that the most prominent antigen in APS is b_2_-GPI, a protein with relatively low affinity towards anionic phospholipids. The importance of antibodies against b_2_-GPI was demonstrated by injection of these antibodies in mice, which resulted in increased thrombus formation when challenged and they showed an increased resorption of fetuses when pregnant. Despite the obvious importance of b_2_-GPI in the pathophysiology of APS, these in vivo experiments did not reveal a physiological function for this protein.

It was already know for a long time that no circulating immune complexes could be detected in plasma of patients with APS, suggesting that the epitope recognized by the auto-antibodies was cryptic. Moreover, different studies have shown that the presence of auto-antibodies against domain I of b_2_-GPI correlates much better with clinical manifestations than auto-antibodies against the whole protein. This assumption was confirmed by the observation that b_2_-GPI can exist in at least two conformations, a circular conformation and a stretched conformation. In plasma, b_2_-GPI predominantly circulates in the circular conformation in which N-terminal domain I interacts with the C-terminal domain V. When b_2_-GPI binds to anionic phospholipids, it is converted into the stretched conformation. This conformation exposes an otherwise cryptic epitope in domain I and the protein can be recognized by the circulating auto-antibodies.

In recent years, novel and exciting data have become available that suggest an important function of this protein in innate immunity. b_2_-GPI was found to scavenge lipopolysaccharide (LPS) and was able to clear unwanted anionic cellular remnants such as microparticles from the circulation. The scavenger function of b_2_-GPI is dependent on the stretched conformation. After binding of LPS, b_2_-GPI is cleared by monocytes. The new indications regarding a possible physiological role of b_2_-GPI could also shed light on the events that cause the formation of auto-antibodies against the protein, and why auto-antibodies against this protein results in an increased risk for thrombo-embolic complications and fetal losses.

## Disclosure of interest

None declared.

